# The Fetters of Organization (Minority Rule)

**Published:** 1906-04

**Authors:** 


### The Fetters of Organization (Minority Rule).

Every day it is becoming more evident that the great majority of the members of the American Medical Association put their heads into a noose when they adopted the present form of organization. The new scheme present superficially an ideal system of well-defined government. But it is not as clear, superficially, why a body of learned men, ostensibly banded together for the benefits of *the whole,* should require such an elaborate adjustment of the voting power.

Theoretically, the new plan promised to develop the unit of medical organization, the county society, and to relieve the annual meetings of the confusion incident to the consideration of business matters in open session. It also assured a substantial increase in membership, with all the benefits that would naturally accrue in revenue and power. Practically, the most tangible result has been to centralize the control of the whole regular profession in the hands of a small coterie of shrewd politicians, many of whom are not medical men at all. The Association membership has increased materially—but without the slightest change in the irrevocable subordination of the many to the dictation of the few.

The condition is intolerable to the individual member who attends the meetings, if he stops to consider the facts. As an individual he has absolutely no more voice in the discussion of questions which arise at meetings of the Association—however faithfully he may attend and punctiliously pay his annual dues—than his brother member of the county society, who may scoff at the idea of joining the National body. As it has worked out, therefore, about the only *bona fide* privilege enjoyed by the individual member is that of wearing a red cross button and of having his name entered on the mailing list of the Association Journal.

The originators of the reorganization plan were bright. In fact, it would be no exaggeration to say that they were cute. They well knew that the county society with its combination of few affiliated and many non-affiliated members of the Association would never be a factor in Association management. The whole scheme is beautiful in its simplicity. Once a year delegates from the county society are elected to the State organization. These delegates from the county society, many of whom are non-members of the National Association, form the House of Delegates of the State Society.

The State House of Delegates elects in turn a certain arbitrary number of national delegates, who form the House of Delegates of the A. M. A. and who alone have the privilege of considering and voting on current Association matters. It can be readily seen how far a cry it is from the individual member who goes to the meetings and the State delegate in the Association House of Delegates, who is nominally supposed to represent him.

From the foregoing it is apparent that non-members and stay-at-homes have more to do with the disposition of issues presented at any meeting than active members in attendance who are naturally interested in questions as brought up. Literally, then, the whole voting power is vested in delegates who hold their positions, not through the influence of those who are alive to Association matters, but through the indifferent votes of those who are not.

Can any one fail to see the pernicious possibilities?

The members who attend and who, from intelligent interest, might prove dangerous to the "Machine," are effectually eliminated. If, perchance, a member wishes to raise an issue, he must pursue a devious course, and either begin his fight in the county society—whose members are apt to be indifferent to questions concerning the National Association—or, if it is relevant, he may possibly secure consideration for it by the section with which he affiliates. If he be successful in the county society, the most he can accomplish is to have the county delegates instructed to bring the matter before the House of Delegates of the State society, and so on; or he may, by dint of strenuous effort, have his section instruct its delegate to submit the matter to the Association House of Delegates. In either event, the actual presentation of the matter must be done by a party who can not be expected to have other than a secondary interest in the subject he presents. From delay or indifference, action or even consideration is pretty sure to be deferred until too late, or until either the issue or its promulgator are dead.

Hence, the more study one gives to the organization of the American Medical Association, as now evolved, the more the conviction will grow that the great body of the members of the A. M. A. have been "gold-bricked" with a finesse of flim-flam that is Chicagoesque, to say the least.

There can not be the slightest doubt that concentration of management was the ulterior motive of the whole project, and that it has been effected far beyond the wildest hopes of the instigators hardly calls for specious argument. If they had been as sincere in their intentions as they would have the profession believe, some adequate provision would have been made for final action on vital questions by the members in attendance at each meetin. As at present constituted, however, there is absolutely no provision for a final review or the exercise of the power of veto, and herein lies the danger.

Assuredly it would have been a satisfactory evidence of good faith if the reorganizers had included some fair arrangement whereby the conclusions of the House of Delegates could be finally acted upon in at least one general session.

Their neglect to provide some such opportunity for the expression of opinions by individual members in attendance is sufficient, in itself, to show underlying motives—sinister beyond doubt.

It is a shame, therefore, that the American medical profession should leave a great organization that should mean so much to science in the hands of a few selfishly ambitious men, who control Association affairs as completely as the Triumvirate ever controlled ancient Rome.

The pity of it is doubly increased, moreover, that an organization so democratically conceived should be in this day of universal science so seriously threatened by so autocratic a miscarriage.—*Editorial in American Medical Journal,* January, 1906.

